# Supporting antidepressant discontinuation using mindfulness plus monitoring versus monitoring alone: A cluster randomized trial in general practice

**DOI:** 10.1371/journal.pone.0290965

**Published:** 2023-09-05

**Authors:** Marloes J. Huijbers, Carolien Wentink, Peter L.B.J. Lucassen, Cornelis Kramers, Reinier Akkermans, Jan Spijker, Anne E.M. Speckens

**Affiliations:** 1 Department of Psychiatry, Radboud University Medical Center, Nijmegen, The Netherlands; 2 Department of Primary and Community Care, Radboud Institute of Health Sciences, Radboud University Medical Center, Nijmegen, The Netherlands; 3 Department of Pharmacology and Toxicology, Radboud University Medical Center, Nijmegen, The Netherlands; 4 Department of Internal Medicine, Radboud University Medical Center, Nijmegen, The Netherlands; 5 IQ Healthcare, Radboud University Medical Center, Nijmegen, The Netherlands; 6 Expertise Centre for Depression, Pro Persona Nijmegen, Nijmegen, The Netherlands; University of Cologne: Universitat zu Koln, GERMANY

## Abstract

Discontinuing antidepressant medication (ADM) can be challenging for patients and clinicians. In the current study we investigated if Mindfulness-Based Cognitive Therapy (MBCT) added to supported protocolized discontinuation (SPD) is more effective than SPD alone to help patients discontinue ADM. This study describes a prospective, cluster-randomized controlled trial (completed). From 151 invited primary care practices in the Netherlands, 36 (24%) were willing to participate and randomly allocated to SPD+MBCT (k = 20) or SPD (k = 16). Adults using ADM > 9 months were invited by GPs to discuss tapering, followed by either MBCT+SPD, or SPD alone. Exclusion criteria included current psychiatric treatment; substance use disorder; non-psychiatric indication for ADM; attended MBCT within past 5 years; cognitive barriers. From the approximately 3000 invited patients, 276 responded, 119 participated in the interventions and 92 completed all assessments. All patients were offered a decision aid and a personalized tapering schedule (with GP). MBCT consisted of eight group sessions of 2.5 hours and one full day of practice. SPD was optional and consisted of consultations with a mental health assistant. Patients were assessed at baseline and 6, 9 and 12 months follow-up, non-blinded. In line with our protocol, primary outcome was full discontinuation of ADM within 6 months. Secondary outcomes were depression, anxiety, withdrawal symptoms, rumination, well-being, mindfulness skills, and self-compassion. Patients allocated to SPD + MBCT (n = 73) were not significantly more successful in discontinuing (44%) than those allocated to SPD (n = 46; 33%), OR 1.60, 95% CI 0.73 to 3.49, p = .24, number needed to treat = 9. Only 20/73 allocated to MBCT (27%) completed MBCT. No serious adverse events were reported. In conclusion, we were unable to demonstrate a significant benefit of adding MBCT to SPD to support discontinuation in general practice. Actual participation in patient-tailored interventions was low, both for practices and for patients. (Trial registration: ClinicalTrials.gov PRS ID: NCT03361514 registered December 2017)

## Introduction

The use of antidepressant medication (ADM) has been rising for many years. It has been estimated that prescription of ADM has doubled between 2003 and 2013 in Western countries [1]. and rates have steadily risen in the Netherlands too [2]. This rise has been mainly explained by an increasing number of long-term users [3]. For example, the median duration of ADM use is more than two years in the UK [4] and over 5 years in the US [5]. Despite being possibly effective in terms of reducing levels of depression and depressive relapse [6, 7], some patients suffer from adverse effects such as sleep disturbance, weight gain, sexual dysfunction and gastrointestinal bleeding [8, 9] or even attempted suicide or self-harm, stroke and epilepsy [10]. Not surprisingly, many patients are unwilling to continue ADM extendedly [11] and prefer psychological treatment [12]. In fact, clinical treatment guidelines typically recommend considering discontinuation of ADM about six months after remission from a depressive episode, up to two years or more for patients with complex or recurrent depression [13, 14] and about 6–12 months after remission of an anxiety disorder [15]. However, discontinuation of ADM seems challenging for patients [16, 17] and clinicians [18].

Systematic reviews have suggested that the most important barriers to discontinuation include fear of relapse or recurrence, actual relapse or recurrence, withdrawal symptoms, insufficient evaluation and monitoring, lack of support, and life circumstances [19, 20]. Stopping ADM is often associated with physical or psychological withdrawal symptoms, including headache, dizziness, electric-shock sensations, sleep disturbance, anxiety and mood swings. To minimize the risk of withdrawal symptoms, it is recommended to taper antidepressants gradually [21–23] and even hyperbolically [24], meaning that dose reductions are large at the beginning of tapering but increasingly small towards the end, for which tapering strips might be of use [25]. Despite such advances, withdrawal symptoms are common and for some patients, they can be long-lasting [26]. In addition, withdrawal symptoms might be misinterpreted as a return of the original symptoms, leading to prolonged and possibly unnecessary treatment with ADM [27]. In turn, this may also have a negative impact on patients’ autonomy and self-esteem, feeling that they are unable to cope without medication [18].

Discontinuation rates range between 40 and 95% when additional support is provided, and may be as low as 6% and 7% when patients only receive the advice to taper by their GP [28]. There appears to be a need for taper support that is insufficiently addressed in current health care, drawing people to online communities for peer support [29]. Patients and general practitioners (GPs) may be waiting for each other to initiate a conversation about tapering [18]. A decision aid may be a useful tool for a balanced decision about tapering [30]. In addition, there has been an upsurge in the development of psychological interventions to aid discontinuation, and these can be helpful [28, 31].

Mindfulness-based cognitive therapy (MBCT) has been hypothesized to support the tapering process [32], and at the same time provide ways to cope with withdrawal symptoms and distress, and prevent depressive relapse [33]. Indeed, in an individual patient data meta-analysis, MBCT has been found to reduce the risk of depressive relapse/recurrence in individuals with remitted depression, compared with maintenance antidepressants [34]. Three studies compared relapse/recurrence rates in patients with remitted recurrent depression after they had tapered ADM after MBCT, compared with maintenance ADM use [35–37], and found little difference. So, MBCT appeared to be a feasible alternative to ADM in all three studies. In another study, however, MBCT followed by ADM discontinuation was compared with MBCT plus maintenance ADM [38]. That comparison clearly showed a disadvantage for those who discontinued ADM, with depressive relapse rates of 54% (if discontinued) versus 39% (if continued) in the intention-to-treat analysis, rising up to 69% and 46% in the per-protocol analysis. In addition, the cessation rate (complete discontinuation within 6 months) was only 53%. This was possibly due to the recruitment strategy (most patients were particularly interested in MBCT, rather than in discontinuing ADM), the intervention itself (using the standard MBCT protocol and providing discontinuation support outside the mindfulness context) and a relatively swift tapering schedule (5 weeks).

The current study aimed to investigate the effectiveness of MBCT added to supported protocolized discontinuation (SPD) compared with SPD alone to successfully discontinue long-term ADM use in primary care. We selected patients interested in discontinuation, enhanced usual care by providing taper support materials to patients and GPs, and offered patients an MBCT intervention with tapering support explicitly embedded within the mindfulness framework. We used a cluster-randomized design to avoid contamination and to simplify research procedures for GPs and mental health nurses. We hypothesized that the MBCT group would have higher rates of succesful discontinuation. In addition, we expected that adding MBCT to SPD would produce more beneficial mental health outcomes across the 12 months follow-up period.

## Materials and methods

### Study design

We performed a cluster-randomized controlled trial in primary care, including patients who had been using ADM for at least nine months who wished to discontinue. Allocation in a 1:1 ratio was performed at the level of the GP practice to avoid contamination and to simplify research procedures for GPs and mental health nurses. Generation of the numbers for the randomization schedule was performed by a statistician (RA), based on block randomization with blocks of two, stratified for region (north, central and east Netherlands) and type of practice (single versus group). Allocation was performed by the researcher (CW) in collaboration with the statistician who consecutively allocated practices to the randomization scheme. Patients, GPs, and the research team were aware of treatment allocation. The mental health nurses were kept blind to allocation to ensure that SPD was offered in the same way to all participants, and both GPs and patients were asked not to reveal this information. The study was approved by the medical-ethical committee Arnhem-Nijmegen (2016–2527) and all patients gave written informed consent before entering the intervention phase of the study.

### Study population and procedure

For a detailed description of the study procedures see the published protocol [39]. The trial was registered in a publicly accessible registry in December 2017 (ClinicalTrials.gov; NCT03361514), inadvertently 10 months after the start of recruitment due to the research team being insufficiently attentive to timely registration. Future trials will be registered prospectively. There were no changes to the design, procedures or outcomes after trial commencement, except for having a 12-month assessment instead of a 15-month assessment. Data collection took place between February 2017 and July 2019. GPs from three regions in the Netherlands (north, middle and east) were systematically retrieved and invited via e-mail. In case of non-response, we tried to reach them by phone at least twice. Participating GPs selected from their electronic database adults receiving antidepressants for the past 9 months or longer. They excluded patients currently treated by a psychiatrist; with substance use disorder; non-psychiatric indication for ADM (e.g. neuropathic pain); previous mindfulness training within the past 5 years; or cognitive or language impairments. GPs were allowed to specify additional reasons for exclusion. Next, GPs invited eligible patients to contact the researchers. Due to the cluster-randomized design, patients were aware of treatment allocation before deciding to participate in the study. Patients were asked to complete online informed consent and questions about baseline characteristics, after which they received a decision aid [30] on whether or not to discontinue ADM [30] to discuss with their GP. If they decided to discontinue, they were provided with a personal tapering schedule of 6 months based on a discontinuation protocol and invited for the baseline interview of the intervention phase of the study. Further assessments were after 6, 9 and 12 months. Qualified research assistants interviewed patients at each assessment with the SCID (Structural Clinical Interview for DSM-IV Axis I Disorders) [40]. Self-report measures were completed online using LimeSurvey.

### Interventions

#### Supported protocolized discontinuation (SPD)

Supportive consultations were offered by mental health nurses who were trained by the coordinating researcher (CW) and they received the information brochure, discontinuation protocol and a short guideline about how to organize consultations (S1 File). Consultations would focus on the topics outlined in the decision aid [30], monitoring of withdrawal effects and discussing relapse prevention strategies. Dependent of the patient’s need, consultations could be planned before, during or after discontinuation, up to three months post-discontinuation. Note however that SPD was optional.

#### Mindfulness based cognitive therapy (MBCT)

MBCT was offered according to the treatment protocol developed for recurrent depression [33], adapted to the specific needs of this group by including psychoeducation on stopping antidepressants, withdrawal effects and anticipatory anxiety. These adaptations were made based on a pilot group conducted before the trial, in which patients expressed a clear wish for additional information about tapering and support from the teacher in that process. Based on their feedback, sessions 1–4 took place weekly, and sessions 5–8 every two weeks, in groups of 8–10 patients. Each regular session lasted 2.5 hours. In addition, a 6-hour silent practice day between session six and seven was offered, in which patients received all mindfulness practices without interaction or group discussions. Home practice took approximately 30–45 min a day using audio-guided practices (provided as online audio files or on USB stick). Teachers were physicians (one GP, two psychiatrists and one medical doctor), complied with the good practice guidelines of the UK Network for Mindfulness Based Teachers [41] and received two supervision sessions per MBCT course with a senior mindfulness trainer and psychiatrist (AS).

#### Safety and monitoring

Patients were allowed to increase the dosage or restart their medication if needed. In case of difficulties during the discontinuation process patients were advised to contact their GP or mental health nurse. Suicidal risk was monitored with a 6-item interview at each assessment [42]. Serious adverse events were monitored at each assessment.

### Primary outcome

Our primary outcome was the proportion of patients who had fully discontinued their ADM within 6 months (yes/no). Use of medication was measured with a diary and questionnaires at each assessment. We checked all diaries to see if participants had fully discontinued at some point between baseline and 6 months.

### Secondary outcomes

#### Clinician-reported outcomes

- Relapse/recurrence of depression and/or anxiety between baseline, 6, 9, and 12 months as assessed with the SCID-I [40].
- Depressive symptoms, assessed with the Inventory of Depressive Symptoms, clinician rated version [43, 44].

#### Self-reported outcomes

- Withdrawal symptoms: Discontinuation-Emergent Signs and Symptoms, DESS [45]
- Anxiety symptoms: State-Trait Anxiety Inventory, STAI [46]
- Ruminative brooding: brooding subscale of the Ruminative Response Scale Extended (RRS-EXT) [47]
- Well-being: Mental Health Continuum, MHC-SF [48]
- Mindfulness skills: Five Facet Mindfulness Questionnaire, FFMQ-SF [49]
- Self-compassion: Self-Compassion Scale, SCS-SF [50]

### Sample size calculation

We expected a discontinuation rate of 15% for patients in the SPD condition and 40% for patients in the SPD + MBCT condition. To detect this difference with a power of 80%, alpha of 0.05 (two-sided), intraclass correlation of 0.05 due to cluster randomization, which was estimated to range between 0.00 and 0.05 in primary care in a previous study [51], with an average of 5 participants per cluster and a minimum of 24 clusters, 59 patients per group would be needed. We used G-power to calculate the uncorrected sample size, after which we adopted the design factor to adjust the required sample size for cluster sampling, using the following formula: *1 + 0*.05 * (n-1) [52]. Then, to account for an expected drop-out rate of 15%, we aimed to include 138 patients in the study. For further details we refer to the published protocol [39].

### Statistical analysis

The clinical outcome data were analysed and reported on both intention-to-treat (ITT) and per-protocol (PP) analysis. The ITT sample included all patients who were willing to attempt tapering. The PP sample included patients who attended at least 4 sessions of MBCT, if allocated to MBCT. We did not include the SPD intervention as criterium for the per-protocol analysis as this intervention was optional. Multilevel analyses were used to account for the hierarchical structure of the data (i.e. multiple assessments nested within patients, patients nested within practices). Multilevel logistic regression models were used to test differences in discontinuation rates between the conditions. Relapse percentages of depression or anxiety disorders were compared with a Chi^2^ test. Multilevel linear regression analyses were used for all continuous outcomes. To test the difference in change over time between the two groups, we used a model with group, time and the group x time interaction. The interaction term tests the difference in change over time between the groups and is reported in S1 Table. We explored whether any of the baseline characteristics were associated with the willingness to taper, regardless of group allocation, using Chi^2^ analyses for categorical variables and independent t-tests for continuous variables. All analyses were performed in SPSS Statistics version 25.0.

## Results

### Participant flow

**Fig 1:**
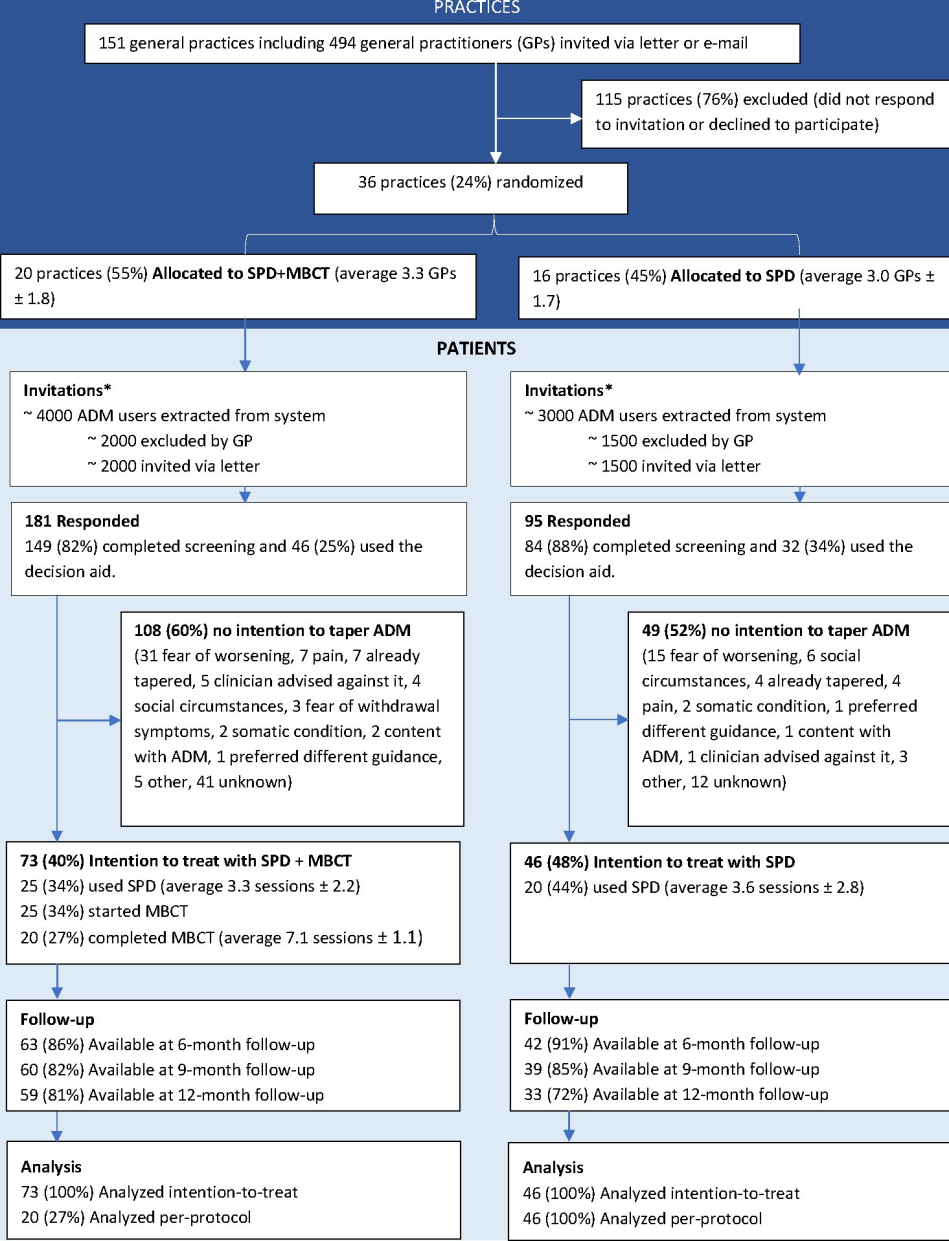
Flow chart of general practices and patients with long-term use of antidepressant medication allocated to supported protocolized discontinuation (SPD) or SPD plus mindfulness-based cognitive therapy (MBCT). Data on the numbers of ADM users, numbers excluded and numbers invited are weighed estimates based on specific data of 12/36 GPs (33%), combined with data on practice size, which were available for all GPs. ADM = antidepressant medication; SPD = supported protocolized discontinuation; MBCT = mindfulness-based cognitive therapy.

Fig 1 illustrates the flow of GPs and participants. About a quarter of invited GPs participated in the study. Although specific data on reasons for exclusion of patients were only available for 12/36 practices, most common reasons for exclusion were currently treated by psychiatrist (38%), pain (15%), already stopped ADM (14%), current depression (7%), somatic comorbidity (5%) and current anxiety disorder (5%). Of the 276 patients who responded to the invitation letter, 157 (57%) decided not to discontinue ADM, and the remaining 119 (43%) were included (SPD+MBCT n = 73; SPD n = 46). Table 1 shows the baseline characteristics of patients in both randomization groups, separately for the ITT sample and those who were not willing to taper. Looking at the entire group of 276 responders, there were no baseline characteristics that seemed associated with the willingness to taper that is, those who proceeded to the intervention phase of the study versus those who dropped out after shared decision making, except for a trend suggesting that individuals who experienced adverse effects when using ADM may have been more willing to taper (54%) than those who did not experience adverse effects (42%).

**Table 1:** Baseline demographic and clinical characteristics of long-term users of antidepressant medication (ADM) in primary care responding to the invitation to participate in a study on discontinuing ADM, cluster-randomized on the level of their general practice to mindfulness-based cognitive therapy (MBCT) plus supported protocolized discontinuation (SPD) or SPD alone.

	MBCT+SPD		SPD	
	Not willing to taper (N = 108)	Intent-to-taper (N = 73)	Not willing to taper (N = 49)	Intent-to-taper (N = 46)
**Female gender, n/n (%)**	79/108 (73)	46/73 (63)	34/49 (69)	31/46 (67)
**Marital status, n/n (%)**				
• **Married/cohabiting**	59/76 (78)	48/73 (66)	24/38 (63)	33/46 (72)
• **Single**	5/76 (7)	10/73 (13)	8/38 (21)	7/46 (15)
• **Divorced**	10/76 (13)	13/73 (18)	4/38 (11)	5/46 (11)
• **Widowed**	2/76 (2)	2/73 (3)	2/38 (5)	1/46 (2)
**Educational level, n/n (%)**				
• **Low**	9/74 (12)	13/73 (18)	5/36 (14)	6/46 (13)
• **Middle**	34/74 (46)	18/73 (25)	14/36 (39)	19/46 (41)
• **High**	31/74 (42)	42/73 (57)	17/36 (47)	21/46 (46)
**Employed, n/n (%)**	42/75 (56)	47/73 (64)	19/38 (50)	28/46 (61)
**Type of ADM, n/n (%)**				
• **Paroxetine**	20/73 (27)	18/73 (25)	9/37 (24)	13/46 (28)
• **Venlafaxine**	7/73 (10)	21/73 (29)	10/37 (27)	8/46 (17)
• **Citalopram**	17/73 (23)	19/73 (26)	7/37 (19)	6/46 (15)
• **Sertraline**	9/73 (12)	7/73 (9)	1/37 (3)	4/46 (9)
• **Escitalopram**	2/73 (3)	0/73 (0)	0/37 (0)	5/46 (11)
• **Other** ^**a**^	18/73 (25)	8/73 (11)	10/37 (27)	9/46 (20)
**Previous tapering attempt “yes”, n/n (%)**	47/73 (64)	48/73 (66)	25/38 (66)	32/46 (70)
**Experiences side effects, n/n (%)**	40/81 (49)	39/70 (56)	18/40 (45)	29/43 (67)
**Age, mean ± SD**	57.8 ± 12.9 (*n = 76*)	53.3 ± 11.8 (*n = 73)*	54.3 ± 14.5 (*n = 39)*	54.7 ± 12.5 (*n = 46)*
**Baseline depression level (PHQ), mean ± SD**	6.5 ± 4.7 (*n = 71)*	5.9 ± 4.8 (*n = 73)*	5.7 ± 5.0 (*n = 38)*	6.2 ± 3.9 (*n = 46)*
**Baseline anxiety (GAD-7), mean ± SD**	4.0 ± 3.4 (*n = 71)*	4.0 ± 3.9 (*n = 73)*	4.2 ± 4.6 (*n = 38)*	3.7 ± 3.0 (*n = 46)*

^a^ Includes amitriptyline, clomipramine, duloxetine, fluoxetine, fluvoxamine, nortriptyline, maprotiline, and mirtazapine. PHQ = patient health questionnaire; GAD-7 = generalized anxiety disorder– 7 items.

### Primary outcome

The primary outcome was available for 117/119 participants (98.3%). In the ITT analysis, 31/71 (44%) of the participants discontinued ADM in the SPD+MBCT group versus 15/46 (33%) in the SPD group: OR 1.60 (95% CI 0.73 to 3.49), corresponding to a number needed to treat of 9. Only 20/73 patients allocated to MBCT (27%) completed the intervention. In the PP analysis, 10/20 (50%) of the participants discontinued ADM in the SPD+MBCT group versus 15/46 (33%) in the SPD group: OR 2.06 (95% CI 0.69 to 6.14), corresponding to a number needed to treat of 6.

Of the 31 patients who discontinued in the SPD+MBCT group, 13 (42%) restarted during the 12-month study period, 11 (35%) did not, and for seven (23%) it was unknown. Of the 15 patients who discontinued in the SPD group, nine (60%) restarted, five (33%) did not, and for one (7%) it was unknown.

### Secondary outcomes

Based on the ITT analysis, relapse into depression or anxiety occurred in 15/73 participants (21%) in the SPD+MBCT group, of whom 6 had anxiety disorder and 9 depressive disorder, and in 5/46 participants (11%) in the SPD group, of whom 1 had anxiety and 4 depressive disorder. Although the confidence interval includes 1.0, the point estimate suggests that patients in the MBCT group are twice more likely to relapse than those in SPD (OR 2.12, 95% CI 0.71 to 6.30).

S1 Table shows that none of the secondary outcomes showed a different trajectory over time between the groups.

### Harms

Aside from expected symptoms related to ADM withdrawal (S2 and S3 Tables), no serious adverse events were reported by participants. One participant reported suicidal thoughts, but not tendencies, at 6-month follow-up. Another participant reported that the assessments evoked stress, after which she decided to withdraw from the study. None of these events required medical help.

## Discussion

### Discontinuation of antidepressants after MBCT

Discontinuation rates at 6-month follow-up were slightly higher in the SPD + MBCT group (44%) than in the SPD group (33%). This difference was smaller than we had hypothesized, which can in part be explained by the SPD group showing a higher discontinuation rate (33%) than we had anticipated (15%). Perhaps this was caused by our selective sample; those who did proceed to the intervention phase of the study were probably motivated to give discontinuation a try. Although the discontinuation rate in the MBCT group (44%) was in line with our a priori expectation (40%), it was at the low end of estimates from a systematic review, in which 40% to 95% of participants were able to discontinue when supported by psychological or psychiatric treatment [28]. In studies focusing specifically on MBCT in recurrent depression, previously reported discontinuation rates varied between 53% [38] and 70–75% [35, 36]. Relatively low discontinuation rates in the current study may be related to the fact that only a minority (27%) adhered to the MBCT protocol in the current study, which is much lower than for MBCT participants in routine clinical practice, estimated between 81–96% [53–55]. Possible reasons for these low participation rates are discussed below. Secondary outcomes did not differ between the groups.

### Participation in the taper support interventions

The current study adopted a comprehensive approach to implementing taper support in primary care. This included an inclusive recruitment strategy in a relatively large number of GP practices, offering a decision aid to discuss advantages and disadvantages of tapering, materials to devise a gradual and patient-tailored tapering schedule, plus additional support from a mental health nurse (i.e. SPD) and a patient-tailored MBCT intervention. Thus, in theory, our approach seemed adequate to reach and support professionals and patients who may have experienced a lack of support in regular healthcare. Nevertheless, despite the perceived need for such support, actual participation in patient-tailored interventions was low, both at the level of the GP practices (only a quarter of the invited practices participated), and at the level of patients of whom only about one tenth responded to the study invitation. This is in line with previous studies that aimed to support tapering in long-term users [4, 56]. Reasons for non-participation for GPs were not systematically collected, but ‘lack of time’ was often mentioned. Reasons for non-participation of non-responding patients were unknown.

Of those who did respond to the invitation, less than half participated in the intervention study. Patients’ reasons for non-discontinuation included fear of recurring symptoms, pain, somatic comorbidity, social circumstances, or clinicians’ advice against tapering. Only a minority of the patients actually reported using the decision aid with their GP, so we do not know to what extent this document could be helpful in making a truly shared and balanced decision about whether or not to taper ADM. Perhaps some of the reasons for not tapering that patients mentioned in the research interviews (e.g. fear of worsening, pain, social circumstances) already shaped their decision before even using the decision aid. Another possible explanation is that the decision aid was insufficiently personalized, despite being co-created with diverse stakeholders to ensure a wide scope of topics. We may need to investigate patient expectations and values and GP’s considerations on an individual level. For example, patients’ beliefs about the causes of their symptoms were not included in the decision aid. If many patients adopted a biomedical framework and believed that their depressive symptoms for example were due to a biochemical imbalance that needs to be restored with medication, the relatively low engagement in tapering is not surprising.

The low participation rate in the MBCT intervention might have been due to our primary focus on withdrawal from ADM, so the resulting sample may have been less “mindfulness-minded” than a self-selecting sample. Perhaps patients who responded to our study invitation were interested in tapering their medication rather than in attending eight weekly 2,5 hour MBCT classes, despite being informed about the time investment of this intervention. Work and travel issues were the most common barriers for participation in MBCT.

### Limitations

Our recruitment strategy was designed to keep the possible selection bias of mindfulness studies at a minimum, thereby increasing the generalizability of the findings. The other side of the coin, however, is that many of those allocated to MBCT did not choose to participate in the intervention after all. Similarly, SPD was used by less than half of the participants. Thus, a major limitation of the current study is that adherence to the interventions was low. Consequently, we are unable to say whether MBCT added to SPD has the potential to significantly increase successful tapering of ADM. The study was underpowered to detect a small difference in discontinuation rates. Another limitation is that we failed to systematically assess reasons for non-participation or non-discontinuation at all stages of the process when implementing our taper support strategies. For example, we do not know why participants did not use the SPD consultations as intended. Furthermore, the partnership between patient and GP was not explicitly targeted, and responsibility for making an appointment to discuss tapering was given to the patients only. In addition, the current study selected patients who all expressed a desire to discontinue their medication. Therefore, we cannot generalize our findings to all patients who are using antidepressant medication. For the aim of this study, however, it seemed most appropriate ‐ both on a practical and ethical level ‐ to select those who are motivated to taper their medication. Finally, a methodological limitation is that we used block sizes of two in our randomization schedule. Although we chose this smaller block size to prevent unequal distribution within cells that were expected to have lower numbers of GP practices due to the stratification variables (region: north, central and east Netherlands; and type of practice: single versus group), this small block size does come with a larger risk of prediction.

### Future directions

Our findings of low engagement raise the question where and how we can find the patients who are ready and willing to taper. Participants did not actively ask for support but were invited in the context of the study. An alternative approach could be to target our interventions at those who actively request taper support. However, from previous studies we know that patients believe that it is the doctor’s responsibility to initiate the conversation about tapering ADM [20]. Without active recommendation by the GP and in absence of consistent guidelines or knowledge about when to start tapering, patients might leave the situation as it is. Therefore, a shared decision making process is essential. This does not have to be limited to GPs, and qualitative studies suggest that there might also be a role for other health care professionals (mental health assistants, psychiatrists or psychologists) or significant others (family or friends) [20]. To promote engagement, fostering the partnership between patients, health care teams and even researchers might be an important step forward, as suggested by a study investigating a coproduction model for opioid tapering [57]. A similar approach will probably be useful for antidepressant tapering too, with a focus on acceptable, feasible and tailored forms of support.

In addition, new forms of support are being developed, such as ADvisor [58], a digital intervention designed as part of the REDUCE programme [59] to support ADM discontinuation, which integrates different kinds of resources that people can use flexibly (including psychoeducation, relapse prevention techniques grounded in MBCT, goal setting exercises based on acceptance and commitment therapy, techniques for managing distress, tools for activity planning and information for family and friends).

Work or travel issues were most often mentioned as barriers to participation. Therefore it would be interesting to include different formats of MBCT, such as stand-alone, blended or therapist-assisted online mindfulness interventions. At the same time, this might challenge the possibly important role of peer support that emerged from the post-MBCT interviews with a subsample of the patients. Video-conferencing programs would overcome the issue of travel time, and may be more appealing for patients with physical problems. In stand-alone interventions, audiovisual materials such as interviews with ‘peers’ who tell about their experiences might help to connect with others’ experiences. Similar adaptations can also be considered for SPD or other forms of monitoring in primary care.

Furthermore, we might consider partial rather than full discontinuation as a valid outcome as well, given the evidence that most withdrawal problems might occur at the lowest doses [24] and there is preliminary evidence for relatively low depressive relapse rates after partial discontinuation [60]. In fact, mindfulness training may even help to be more flexible, self-compassionate and open-minded towards using different treatments to manage depressive symptoms [32].

## Conclusions

The current study did not demonstrate a significant benefit of adding MBCT to SPD to support discontinuation in general practice. Considering the low engagement in this specific context, this should not be taken as a generalized negative recommendation against MBCT to support ADM discontinuation, but it does raise some important questions about recruitment strategies, shared decision making, and (alternative) methods of treatment delivery.

Given the apparent difficulties with tapering ADM, it seems essential that those who prescribe them do so very carefully, preferably for a restricted period of time, revising patients regularly, and taking an active role in informing the patient about possible tapering (ideally already before initiation of ADM use), and supporting the process of discontinuation. The mental health nurse might play a role in the latter. In a recent scoping review, deprescribing intervention activities included identification of appropriate patients for deprescribing, patient education, GP education, and development and use of a tapering schedule [61]. This review also emphasized the role of practice staff in facilitating deprescribing in primary care. Clinical guidelines should be updated to provide doctors with more information on when and how to taper ADM. Future approaches might include stand-alone, blended or therapist-assisted online (mindfulness) interventions to increase accessibility. This might also serve those who prefer individual treatment.

## Supporting information

S1 File Guideline for supporting patients to discontinue their antidepressant medication. (PDF)

S2 File CONSORT extension cluster trials. (DOCX)

S1 Table Dimensional outcomes of long-term users of antidepressant medication in primary care, discontinuing with either supported protocolized discontinuation (SPD) plus mindfulness-based cognitive therapy (MBCT) or with SPD alone, at 0, 6, 9 and 12 months. (PDF)

S2 Table Numbers of patients suffering from withdrawal symptoms (scoring 2 “a little burdensome” or 3 “very burdensome” on the Discontinuation-Emergent Signs and Symptoms (DESS) questionnaire after intending to discontinue antidepressant medication (intention-to-treat) with supported protocolized discontinuation (SPD) or SPD plus mindfulness-based cognitive therapy (MBCT) at baseline, 6, 9 and 12 months. (PDF)

S3 Table Numbers of patients suffering from withdrawal symptoms (scoring 2 “a little burdensome” or 3 “very burdensome” on the Discontinuation-Emergent Signs and Symptoms (DESS) questionnaire after *actually discontinuing* antidepressant medication with supported protocolized discontinuation (SPD) or SPD plus mindfulness-based cognitive therapy (MBCT) at baseline, 6, 9 and 12 months. (PDF)
