# Supplementary material for: Supporting antidepressant discontinuation using mindfulness plus monitoring versus monitoring alone: A cluster randomized trial in general practice
Source: PLoS One. 2023 Sep 5;18(9):e0290965. doi: 10.1371/journal.pone.0290965 (PMC10479886; doi:10.1371/journal.pone.0290965)
Supplement: S1 File — (PDF) [file pone.0290965.s001.pdf]

Supplementary file S1. Guideline for supporting patients to discontinue their antidepressant medication.

Preparations:

Patient has been provided with the decision aid on tapering antidepressants (Wentink, Huijbers et al. 2019) and has devised a personal tapering schedule together with the general practitioner.

The mental health nurse (or health care professional who will carry out the monitoring) has knowledge about withdrawal symptoms, possible tapering schedules and available doses, including tapering strips. Whenever available, the mental health nurse should be provided with the patients' preferences and expectations as detailed in the decision aid, and should have the personal tapering schedule as devised with the GP.

Planning:

Ideally, monitoring consultations take place before, during and after discontinuation, up to three months post-discontinuation. Particular attention should be paid around the time dose approaches 0 mg.

Content of the monitoring consultations:

Before tapering:	• Confirm that tapering schedule is feasible, discuss possible adaptations (e.g. slowing down)<br>• Check which aspects of the decision aid are most important to the patient<br>• Discuss their expectations and needs<br>• Discuss which possible barriers are expected and which opportunities or abilities can be used to facilitate discontinuation
During tapering:	• Monitoring of mental and physical health<br>• Review of the tapering process: is patient still following the schedule?<br>• Installing a relapse prevention plan: what signals of deterioration should particularly receive attention, and what actions can be taken to prevent further deterioration?<br>• Helping patients cope with withdrawal symptoms (if any)
After tapering:	• Helping patients cope with symptoms of depression or anxiety, or resurfacing emotions (some of which may have been suppressed during ADM use)<br>• Discuss life circumstances: which aspects are difficult, which are supporting?

Wentink, C., et al. (2019). "Enhancing shared decision making about discontinuation of antidepressant medication: a concept-mapping study in primary and secondary mental health care." British Journal of General Practice **69**(688): e777-e785.
