# Supplementary material for: Supporting antidepressant discontinuation using mindfulness plus monitoring versus monitoring alone: A cluster randomized trial in general practice
Source: PLoS One. 2023 Sep 5;18(9):e0290965. doi: 10.1371/journal.pone.0290965 (PMC10479886; doi:10.1371/journal.pone.0290965)
Supplement: S1 Table — (PDF) [file pone.0290965.s003.pdf]

Supplementary Table S2. Dimensional outcomes of long-term users of antidepressant medication in primary care, discontinuing with either supported protocolized discontinuation (SPD) plus mindfulness-based cognitive therapy (MBCT) or with SPD alone, at 0, 6, 9 and 12 months.

**Variable**	**SPD + MBCT (allocated n=73)**				**SPD (allocated n=46)**				**Statistical analysis**			
	0 Months<br>Mean ± SD (n)	6 Months<br>Mean ± SD (n)	9 Months<br>Mean ± SD (n)	12 months<br>Mean ± SD (n)	0 Months<br>Mean ± SD (n)	6 Months<br>Mean ± SD (n)	9 Months<br>Mean ± SD (n)	12 months<br>Mean ± SD (n)	Time x group fixed effect	Δ effect MBCT compared to SPD 0-6 mo	Δ effect MBCT compared to SPD 0-9 mo	Δ effect MBCT compared to SPD 0-12 mo
Depressive symptoms (IDS-C)	6.26 ± 5.50 (73)	9.15 ± 6.18 (62)	8.83 ± 6.23 (52)	8.70 ± 5.47 (53)	5.35 ± 4.22 (46)	9.88 ± 7.50 (41)	9.31 ± 5.20 (32)	9.71 ± 6.33 (31)	F(3, 281) = 0.83; *p*= .478	-1.79 (CI -4.45, 0.86)	-1.26 (CI -4.11, 1.60)	-1.97 (CI -4.84, 0.90)
Withdrawal symptoms (DESS)	0.69 ± 0.50 (69)	0.64 ± 0.46 (58)	0.67 ± 0.47 (55)	0.62 ± 0.46 (54)	0.67 ± 0.36 (42)	0.71 ± 0.39 (39)	0.58 ± 0.31 (35)	0.62 ± 0.38 (31)	F(3, 269) = 2.93; *p*= .069	-0.12 (CI -0.25, 0.01)	0.06 (CI -0.08, 0.19)	0.01 (CI -0.13, 0.15)
Anxiety – state (STAI-S)	2.05 ± 0.57 (69)	2.07 ± 0.56 (58)	2.11 ± 0.57 (55)	1.98 ± 0.55 (54)	2.02 ± 0.47 (42)	2.12 ± 0.47 (39)	2.06 ± 0.45 (36)	2.20 ± 0.53 (31)	F(3, 276) =2.36; *p*= .072	-0.11 (CI -0.33, 0.10)	-0.00 (CI -0.22, 0.22)	**-0.27 (CI -0.50, -0.04) *p*=.02**
Anxiety – trait (STAI-T)	2.11 ± 0.51 (69)	2.12 ± 0.45 (58)	2.12 ± 0.46 (55)	2.09 ± 0.48 (54)	2.15 ± 0.44 (42)	2.23 ± 0.46 (39)	2.17 ± 0.44 (36)	2.24 ± 0.49 (31)	F(3, 273) =1.03; *p*= .380	-0.10 (CI -0.26, 0.06	-0.02 (CI -0.19, 0.15	-0.13 (CI -0.30, 0.04
Ruminative brooding (RRS brooding)	9.83 ± 3.15 (69)	9.81 ± 2.77 (57)	10.04 ± 3.08 (55)	9.81 ± 2.86 (54)	9.83 ± 2.94 (41)	9.92 ± 2.63 (39)	9.74 ± 3.26 (35)	10.10 ± 3.19 (31)	F(3, 270) =0.88; *p*= .454	-0.39 (CI -1.40, 0.63)	0.24 (CI -0.80, 1.28)	-0.56 (CI -1.63, 0.51)
Mental well-being (MHC-SF)	2.65 ± 1.08 (69)	2.72 ± 0.99 (57)	2.48 ± 1.05 (55)	2.61 ± 1.00 (54)	2.70 ± 1.10 (42)	2.58 ± 1.10 (39)	2.66 ± 1.03 (35)	2.65 ± 1.08 (31)	F(3, 265) =1.73; *p*= .160	0.22 (CI -0.07, 0.51)	-0.13 (CI -0.43, 0.17)	0.04 (CI -0.27, 0.35)
Mindfulness (FFMQ-SF)	81.35 ± 11.97(69)	81.16 ± 10.40(57)	80.42 ± 11.21(55)	80.04 ± 11.39(54)	82.29 ± 9.74 (42)	78.21 ± 11.82(39)	80.26 ± 13.24(35)	81.19 ± 13.76(31)	F(3, 273) =2.06; *p*= .106	**4.45 (CI 0.44, 8.47), *p*=.03**	0.84 (CI -3.27, 4.95)	-0.02 (CI -4.27, 4.22)
Self-compassion (SCS-SF)	25.51 ± 6.41 (67)	25.52 ± 6.23 (56)	25.49 ± 6.04 (55)	25.32 ± 7.00 (54)	26.45 ± 5.84 (41)	25.47 ± 6.71 (39)	25.59 ± 7.37 (35)	25.77 ± 7.48 (31)	F(3, 267) =0.55; *p*= .647	1.32 (CI -0.84, 3.47)	1.06 (CI -1.14, 3.26)	0.93 (CI -1.33, 3.20)
