# Supplementary material for: Supporting antidepressant discontinuation using mindfulness plus monitoring versus monitoring alone: A cluster randomized trial in general practice
Source: PLoS One. 2023 Sep 5;18(9):e0290965. doi: 10.1371/journal.pone.0290965 (PMC10479886; doi:10.1371/journal.pone.0290965)
Supplement: S3 Table — (PDF) [file pone.0290965.s005.pdf]

Supplementary Table S4. Numbers of patients suffering from withdrawal symptoms (scoring 2 "a little burdensome" or 3 "very burdensome" on the Discontinuation-Emergent Signs and Symptoms (DESS) questionnaire after *actually discontinuing* antidepressant medication with supported protocolized discontinuation (SPD) or SPD plus mindfulness-based cognitive therapy (MBCT) at baseline, 6, 9 and 12 months.

	**MBCT + SPD (tapered N = 31)**				**SPD (tapered N = 15)**			
Symptom domain	*Baseline (n=30)*	*6 mo (n=24)*	*9 mo (n=25)*	*12 mo (n=26)*	*Baseline (n=12)*	*6 mo (n=14)*	*9 mo (n=13)*	*12 mo (n=10)*
balance	10 (33%)	4 (17%)	4 (16%)	4 (15%)	5 (42%)	3/12 (21%)	3 (23%)	2 (20%)
gastrointestinal	15 (50%)	19 (79%)	8 (32%)	9 (35%)	4 (33%)	4 (29%)	3 (23%)	6 (60%)
flu-like	23 (77%)	19 (79%)	21 (84%)	16 (62%)	9 (75%)	10 (71%)	11 (85%)	9 (90%)
sensory	12 (40%)	9 (38%)	9 (36%)	10 (39%)	7 (58%)	4 (29%)	6 (46%)	3 (30%)
sleep	15 (50%)	15 (63%)	13 (52%)	14 (54%)	9 (75%)	7 (50%)	7 (54%)	6 (60%)
affective	17 (57%)	20 (83%)	17 (68%)	16 (62%)	10 (83%)	10 (71%)	10 (77%)	7 (70%)
other	17 (57%)	12 (50%)	13 (52%)	11 (42%)	10 (83%)	8 (57%)	9 (69%)	7 (70%)

Notes. Numbers of available participants vary per time point due to missing data. Patients who report a score of 2 a little burdensome" or 3 "very burdensome" on the DESS on at least one item of the concerning subscale were included in the numbers presented. The category "other" includes muscle stiffness, cramps or spasms, restless legs, shaking, uncontrollable mouth or tongue movement, speech or articulation problems, increased saliva, and shortness of breath.
